# Interstitial Foci Expression of Indoleamine 2,3-Dioxygenase 1: A Potential Biomarker for Kidney Transplant Rejection

**DOI:** 10.3390/jcm13144265

**Published:** 2024-07-22

**Authors:** Krzysztof Wiśnicki, Piotr Donizy, Magdalena Kuriata-Kordek, Izabella Uchmanowicz, Justyna Zachciał, Agnieszka Hałoń, Dariusz Janczak, Mirosław Banasik

**Affiliations:** 1 Department of Nephrology and Transplantation Medicine, Wroclaw Medical University, 50-367 Wroclaw, Poland; magdalena.kuriata-kordek@umw.edu.pl; 2 Department of Clinical and Experimental Pathology, Wroclaw Medical University, 50-367 Wroclaw, Poland; piotr.donizy@umw.edu.pl (P.D.); agnieszka.halon@umw.edu.pl (A.H.); 3 Department of Nursing and Obstetrics, Wroclaw Medical University, 50-367 Wroclaw, Poland; izabella.uchmanowicz@umw.edu.pl (I.U.); justyna.zachcial@umw.edu.pl (J.Z.); 4 Department of Vascular, General and Transplantation Surgery, Wroclaw Medical University, 50-367 Wroclaw, Poland; dariusz.janczak@umw.edu.pl

**Keywords:** kidney transplantation, indoleamine 2,3-dioxygenase 1 (IDO1), graft rejection, biomarker, immunohistochemistry, antibody-mediated rejection (AMR), T-cell-mediated rejection (TCMR)

## Abstract

(1) **Background**: Kidney transplantation is the best therapy for patients with end-stage renal disease, but the risk of rejection complicates it. Indoleamine 2,3-dioxygenase 1 (IDO1), an enzyme involved in immune response modulation, has been suggested to play a role in transplant immunological injury. The aim of the study was to explore the expression of IDO1 in the interstitial foci of transplanted kidneys and its potential association with rejection episodes. (2) **Methods**: This retrospective study analysed kidney transplant biopsies from 121 patients, focusing on IDO1 expression in interstitial foci. Immunohistochemistry was used to detect IDO1, and patients were categorised based on IDO1 presence (IDO1-IF positive or negative). The incidence of rejection was compared between these groups. (3) **Results**: Patients with IDO1 expression in interstitial foci (IDO1-IF(+)) exhibited higher incidences of rejection 46/80 (57.5%) vs. 10/41 (24.34%) patients compared to IDO1-IF(−) patients, which was statistically significant with *p* = 0.0005. The analysis of antibody-mediated rejection showed that IDO1-IF(+) patients developed AMR at 12/80 (15%), while only 1 IDO1-IF(−) negative patient did (2,44%), with *p* = 0.035. T-cell-mediated rejection was also more common in IDO1-IF(+) patients 43/80 (53.75%) than in IDO1-IF(−) patients 7/41 (17.07%), with *p* = 0.0001. (4) **Conclusions**: IDO1 expression in interstitial foci of renal transplant biopsies is associated with a higher incidence of rejection, suggesting that IDO1 could serve as a potential biomarker for transplant rejection. These findings highlight the importance of IDO1 in immune regulation and its potential utility in improving the management of kidney transplant recipients.

## 1. Introduction

Kidney transplantation is the indisputable gold standard treatment for end-stage renal disease and significantly improves lifespan and patients’ quality of life [1,2,3,4]. Despite advancements in immunosuppressive therapy, particularly those targeting T-cell function, the long-term survival of transplanted kidneys is still a significant challenge due to rejection risk [5,6,7,8]. This calls for immediate and effective medical interventions, typically involving immunosuppressive medications, including corticosteroids and anti-rejection drugs. The complexity of immune response modulation in kidney transplantation requires a delicate balance to prevent rejection while avoiding excessive suppression of the recipient’s immune system.

In this area, the role of indoleamine 2,3-dioxygenase 1 (IDO1), an intracellular enzyme that catalyses the first step in the kynurenine pathway, could be a potential turning point [9,10,11]. The function of IDO1 leads to the depletion of tryptophan and increased kynurenine metabolites, suppressing cell division and triggering apoptosis in effector T cells, simultaneously enhancing the function of regulatory T cells. This fact identifies IDO1 as a pivotal player in regulating immune responses in the context of kidney transplantation [12,13,14,15]. Indoleamine 2,3-dioxygenase expression in different kidney compartments, particularly in interstitial foci, still needs to be sufficiently explored. Given the central role of antibody-mediated rejection in transplant failure and the importance of both cellular and humoral immune responses in graft survival, a comprehensive understanding of the role of IDO1 in these various compartments is crucial [16,17].

This study assesses IDO1 expression in the interstitial foci of renal transplant biopsies. By examining the association of IDO1 expression in IF with various clinical parameters, including rejection, we aim to clarify the varied role of IDO1 in kidney transplantation. This inquiry could pave the way for new methods in the management and prognosis of kidney transplant recipients, enhancing graft survival and patient outcomes.

## 2. Materials and Methods

### 2.1. Patients and Sample Collection

This research conducted a retrospective analysis of 121 renal transplant recipients in the initial investigation. These patients experienced progressive graft dysfunction and were hospitalised at the Clinical Department of Nephrology and Transplantation Medicine of the University Clinical Hospital in Wroclaw between August 2011 and June 2016. Routine medical provision necessitated graft biopsies due to renal function deterioration, defined as an increase in serum creatinine level by 0.3 mg/dL or more. As part of the regular healthcare practice, serum creatinine levels were monitored quarterly in the outpatient clinic. If there was a noted elevation in the creatinine levels, it triggered an action of conducting a biopsy. Initially conducted to assess IDO1 expression in the tubular epithelium (TE), this study further analysed these biopsies, including IDO1 expression in interstitial foci (IF). This expanded analysis provides a more comprehensive understanding of IDO1 expression in the context of renal transplant dysfunction.

### 2.2. IDO1 Expression Analysis

**Figure 1:**
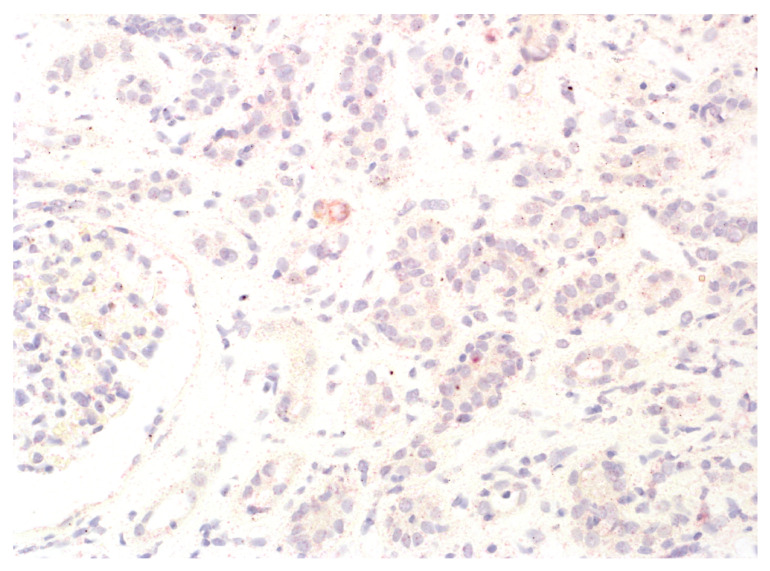
Lack of IDO1 reactivity in the interstitium.

**Figure 2:**
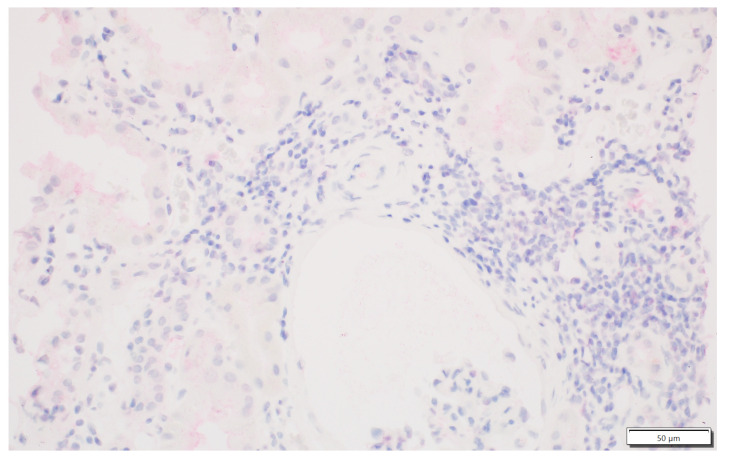
No IDO1 expression in the interstitial immune cells. Focal IDO1 immunoreactivity in tubular epithelium with moderate to severe atrophy.

**Figure 3:**
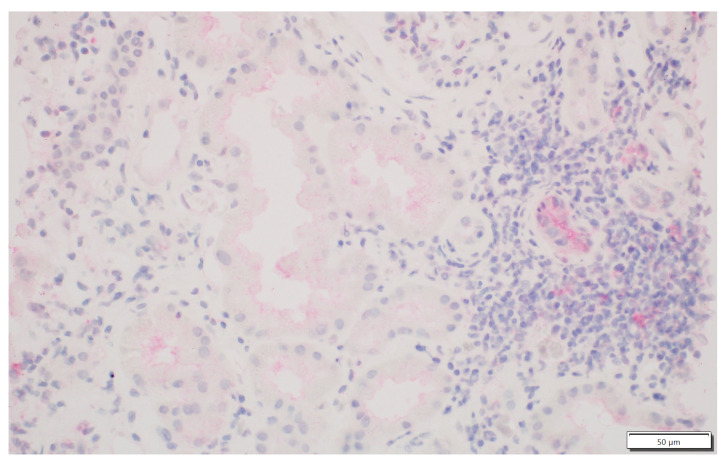
Focal presence of IDO1-positive interstitial immune cells.

**Figure 4:**
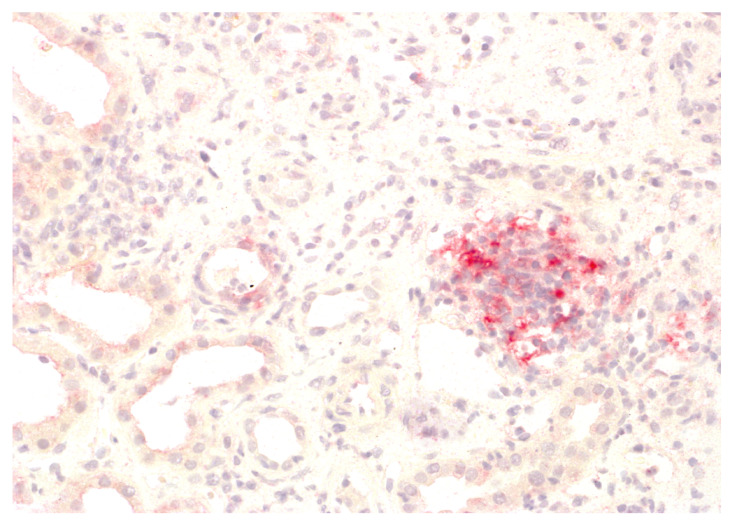
Presence of IDO1 expression in the interstitial immune cells.

We analysed the expression of IDO1 in interstitial foci. The scoring system for IDO1 expression in interstitial foci was maintained on a binary scale, with ‘0’ indicating no IDO1 presence and ‘1’ indicating any presence of IDO1. This binary approach was adopted to assess the potential association of IDO1 presence in interstitial foci with graft rejection. Immunohistochemistry analyses were performed on tissue sections using primary 1F8.2 antibodies against IDO1 (Sigma-Aldrich, Burlington, MA, USA) [18]. The epitope retrieval and staining procedures, including EnVision Target Retrieval Solution (Agilent Technologies, Inc., Santa Clara, CA, USA) and the Autostainer Link 48 (Agilent Technologies, Inc., Santa Clara, CA, USA), were consistent with the earlier study [19,20,21]. It also applies to the detection system, Liquid Permanent Red (Agilent Technologies, Inc., Santa Clara, CA, USA) [22]. The system divided the division into two groups for research: the IDO1-IF(+) positive and the IDO1-IF(−) negative groups. This facilitated a comparative analysis between these groups regarding rejection in transplanted kidneys, causes of chronic renal failure, post-transplantation treatment and a comparison of patient characteristics within each group. The distinction between interstitial immune cells and tubular epithelium, which we focused on in our previous study, was based on detailed histopathologic analyses performed by experienced renal pathologists (PD and AH) [21]. Tubules are structures with lumen, and interstitial immune cells are dispersed and do not form tubular structures. To better showcase the process, Figure 1 shows no IDO1 expression in interstitial foci, and Figure 2 displays IDO1 activity in tubular epithelium in comparison, while Figure 3 and Figure 4 show IDO1 immunoreactivity in interstitial foci.

### 2.3. Sensitivity and Specificity of the Research

To determine the diagnostic accuracy of the enzyme indoleamine 2,3-dioxygenase (IDO1) in predicting kidney allograft rejection, we calculated the sensitivity and specificity of IDO1 detection in biopsy samples. We analysed 121 patients, among whom 56 experienced rejection. Within the IDO1 positive group (80 patients), 46 had rejection, classifying these cases as true positives (TP). In the same group, 34 patients did not have rejection, classifying them as false positives (FP). Among the IDO1 negative group (41 patients), 10 had rejection, classifying these cases as false negatives (FN), and 31 did not have rejection, classifying them as true negatives (TN). Sensitivity, the proportion of actual positives correctly identified by the test, was calculated by dividing the number of true positives (46) by the sum of true positives and false negatives (46 + 10), yielding a sensitivity of approximately 82.1%. Specificity, the proportion of actual negatives correctly identified, was determined by dividing the number of true negatives (31) by the sum of true negatives and false positives (31 + 34), resulting in a specificity of approximately 47.7%. These calculations underscore the test’s ability to identify rejection cases accurately while highlighting areas for improvement in ruling out non-rejection cases.

### 2.4. Statistical Instruments and Programs Utilised in Analysing Data

The statistical analysis was performed using Statistica version 14.1.0 (TIBCO Software, Warsaw, Poland) and R version 4.3.2 (R Foundation for Statistical Computing, Vienna, Austria). The Student *t*-test, Mann–Whitney U, and chi-squared tests were applied appropriately. A *p*-value below 0.05 was considered significant. The dataset was expanded to include information on IDO1 presence in interstitial foci alongside the TE data from the 121 kidney transplant patients.

### 2.5. Ethical Considerations

The original ethical approval granted by the Research Ethics Board of Wroclaw Medical University, under reference number KB 628/2021, entirely covered the extended aspects of this research. Informed consent, previously obtained from all participants, remained valid and ensured ongoing compliance with ethical standards, negating the need for additional consent gathering. It is important to emphasise that our investigation did not increase the risk to any patient involved. Considering that kidney transplant recipients frequently have biopsies because of declining graft function, our study was conducted in strict adherence to existing medical guidelines [23,24,25]. This approach guaranteed that no extra risks were imposed on the patients beyond what is typically expected during the assessment of transplant results.

## 3. Results

### 3.1. Characteristics of Patients

Table 1 presents the primary characteristics of the patients. The data are organised according to indoleamine 2,3-dioxygenase (IDO1) expression in interstitial foci. None of the parameters showed a statistically significant difference between the two groups.

Table 2 shows the immunosuppression approaches following kidney transplantation, as categorised by patients, in relation to the presence of indoleamine 2,3-dioxygenase (IDO1) in interstitial foci. None of the patients received anti-thymocyte globulin as immunosuppression induction.

### 3.2. Rejection Observed in Patients Displaying IDO1 Expression in Interstitial Foci

The analysis of patients with and without IDO1 expression in interstitial foci revealed noteworthy trends in rejection episodes. Among the patients showing IDO1 expression in interstitial foci (IDO1-IF(+) positive), a higher incidence of rejection was observed compared to those without such expression (IDO1-IF(−) negative). In the IDO1-IF(+) positive group, rejection was observed in 57.5% of cases. Conversely, in the IDO1-IF(−) negative group, rejection was noted in 24.34% of subjects. The noted disparity in the incidence of rejection was found to be statistically significant as indicated by a *p*-value of 0.000546. These results suggest a potential association between the presence of IDO1 in interstitial foci and increased rates of graft rejection.

To support our findings, the logistic regression analysis, which takes into account various demographic and clinical variables simultaneously, was performed. The detailed outcomes of this analysis are illustrated in Table 3.

### 3.3. Analysis of Antibody-Mediated Rejection (AMR)

Upon investigating the incidence of antibody-mediated rejection (pure and mixed), it was observed that patients expressing IDO1 in interstitial foci had a higher rate of AMR than those who did not. In the group positive for IDO1 in interstitial foci (IDO1-IF(+) positive), 53.75% exhibited AMR. In contrast, only 2.44% of patients in the IDO1-IF(−) negative group encountered antibody-mediated rejection. Notably, the difference between these two groups was statistically significant as indicated by a *p*-value of 0.03. This observation suggests a potential association between the expression of IDO1 in interstitial foci and the propensity for AMR.

### 3.4. T-Cell-Mediated Rejection (TCMR) Analysis

Similarly, examining T-cell-mediated rejection (pure and mixed) revealed a significant trend. Patients positive for IDO1 in interstitial foci (IDO1-IF(+)) had a higher incidence of TCMR (both pure and mixed) compared to those lacking IDO1 expression in these areas. Accordingly, 43 out of 80 (53.75%) IDO1-IF(+) positive patients and 7 out of 41 (17.07%) IDO1-IF(−) negative patients experienced TCMR. The observed difference was statistically significant as indicated by a *p*-value of 0.0001. This suggests that the presence of IDO1 in interstitial foci may be associated with a heightened risk of TCMR.

### 3.5. Pure Types of Rejection

When focusing specifically on pure forms of rejection (AMR and TCMR), it was found that patients expressing IDO1 in interstitial foci had higher incidences of both types. Pure TCMR exhibited a statistically significant difference between the IDO1-IF(+) and IDO1-IF(−) groups. In the IDO1-IF(+) group, 33 out of 41 patients (41.25%) experienced this type of rejection, compared to 7 out of 41 patients (17.07%) in the IDO1-IF(−) group. This discrepancy was statistically significant, with a *p*-value of 0.007. However, there was no statistically significant difference between the two groups regarding pure AMR. In the IDO1-IF(+) positive group, only 2 out of 80 patients (2.5%) experienced this type of rejection, while in the IDO1-IF(−) negative group, only 1 out of 41 patients (2.44%) did. The *p*-value of 0.983711 indicates that this difference is not statistically significant.

A summary of the influence of IDO1 on different types of rejection is presented in Table 4.

### 3.6. Proteinuria: Another Indication for Biopsy

Out of the 121 patients assessed, 74 were found to have proteinuria, an additional factor that necessitated a biopsy. Among the 80 patients who were positive for IDO1 in interstitial foci (IDO1-IF(+) positive), 44 (55.00%) exhibited proteinuria. Contrarily, in the group of 41 patients who did not show IDO1 expression (IDO1-IF(−) negative), 31 (75.6%) had proteinuria. The statistical analysis revealed a notable distinction between these two groups, supported by a *p*-value of 0.027.

### 3.7. Time Elapsed between Transplantation and Biopsy

Upon examining the duration from transplantation to biopsy for all patients, the mean time was 244 weeks. The IDO1-IF(−) negative group had a mean biopsy time of 218 weeks, while the IDO1-IF(+) positive group had a mean time of 257 weeks. The timing disparity between these two groups was statistically insignificant as denoted by a *p*-value of 0.24.

### 3.8. Delayed Graft Function

Taking the delayed graft function, the inability of the graft to start functioning for a week after transplantation, into consideration, showed no significant difference between the IDO1-IF(+) positive and IDO1-IF(−) negative group [26].

### 3.9. Creatine Level

For creatine levels on the day of the biopsy, there was no significant difference between the IDO1-IF(+) positive (M = 2.57, SD = 2.66) and IDO1-IF(−) negative (M = 2.87, SD = 1.96) groups, t = 0.99839, *p* = 0.160059.

## 4. Discussion

This study expands upon our previous work on IDO1 expression in kidney transplantation and explores its expression in interstitial foci and its relationship with rejection episodes [21]. The results underline a significant variance in the role of IDO1 depending on its location within the kidney.

The presence of IDO1 in the tubular epithelium (TE) has been consistently associated with a protective role against rejection as supported by our earlier findings and [12,21,27,28,29,30]. Previously, we showed that the expression of IDO1 in the tubular epithelium was linked to a lower rejection rate [21]. In this regard, of 76 patients who exhibited IDO1 expression (IDO1(+) positive), only 25 (32.9%) underwent rejection, while in the IDO1(−) negative group, 28 out of 45 (62.2%) experienced rejection. The mechanism is thought to involve the degradation of tryptophan, leading to an immunosuppressive environment that restrains T-cell-mediated rejection [31,32]. Other studies show that the upregulation of IDO1 using antigen-presenting cells could be a viable approach to avoiding graft-versus-host disease [33]. Moreover, IDO1 has been proven crucial in maintaining wild-type mesenchymal stem cell treatment in allograft recipients [34].

In contrast, the existence of IDO1 in interstitial foci paints a different picture. Our study found that in the group of patients with IDO1 expression in interstitial foci (IDO1-IF(+) positive), the rate of rejection was higher compared to the group without such expression (IDO1-IF(−) negative). Specifically, in the IDO1-IF(+) positive group, 46 out of 80 individuals, or 57.5%, experienced rejection. On the other hand, in the IDO1-IF(−) negative group, rejection occurred in 10 out of 41 individuals, which is 24.34%. This result is fascinating and somewhat unexpected, considering the immunosuppressive role of IDO1 we formerly demonstrated in our paper. The difference was relevant to other, specific forms of rejection.

One possible explanation could be the local microenvironment in interstitial foci, which might influence the function of IDO1 differently compared to the tubular epithelium. Alternatively, IDO1 expression in interstitial foci might respond to inflammatory processes associated with rejection rather than a causative factor. An increase in IDO1 activity could indicate that the immune system is attempting to foster tolerance and minimise rejection chances. Suarez and colleagues discovered a link between serum IDO1 activity and an increased risk of rejection following heart transplantation [35]. Furthermore, Weng and the team found that IDO1 expression in peripheral blood was linked to more severe liver transplant rejection in rats [36]. Lastly, Halloran and associates found a correlation between IDO1 and antibody-mediated rejection. The Trifecta Study emphasises the importance of molecular markers such as IDO1 in understanding and monitoring kidney transplant rejection. The strong correlation between IDO1 expression and elevated donor-derived cell-free DNA levels during rejection episodes suggests that IDO1 could be a valuable biomarker for non-invasive diagnostic strategies in kidney transplantation [37]. Further research is needed to elucidate these observations’ underlying mechanisms and implications.

In addition to its role in organ transplantation, IDO1 has also been extensively studied in the context of cancer. IDO1 is a heme enzyme that catalyses the oxidation of L-tryptophan [38]. It has been found to play a pivotal role in cancer immune escape by catalysing the initial step of the kynurenine pathway [39]. Overexpression of IDO1 is associated with poor prognosis in various cancers [38].

The function of IDO1 in cancer is thought to be related to its immunosuppressive properties. The depletion of tryptophan and the increase in kynurenine, a product of tryptophan catabolism, exert essential immunosuppressive functions by activating T regulatory cells and myeloid-derived suppressor cells, suppressing the functions of effector T and natural killer cells and promoting the neovascularisation of solid tumours [39]. This creates an environment that allows cancer cells to evade the immune system.

Several studies have demonstrated that IDO1 is highly expressed in multiple types of human cancer [40,41]. For instance, a comprehensive analysis of the predictive value and immunological role of the IDO1 gene in pan-cancer revealed that IDO1 has abnormal expression in several malignancies and is related to the prognosis of various types of cancer [42]. Furthermore, IDO1 expression was connected to the tumour mutational burden, microsatellite instability, mismatch repair, drug sensitivity, immune cells infiltrating, and the tumour immune microenvironment across various cancer types [42,43,44,45,46]. Targeting the IDO1 pathway has emerged as a promising therapeutic strategy in cancer immunotherapy. Various approaches, including peptide vaccines, expression inhibitors, enzymatic inhibitors, and effector inhibitors, have been explored [39].

The role of IDO1 in both cancer and organ transplantation appears to be linked to its immunomodulatory functions [47,48,49]. In the context of cancer, IDO1 is involved in suppressing effector T and NK cells and activating regulatory T (Treg) cells and myeloid-derived suppressor cells (MDSCs) [15,50,51]. It also plays a key role in promoting tumour neovascularisation by modulating the expression of interferon-γ (IFN-γ) and interleukin-6 (IL-6) [47,48]. Additionally, IDO1 plays a role in creating resistance to immune checkpoint inhibitors. Using an IDO1 inhibitor in conjunction with checkpoint inhibitors is considered a potential new approach in cancer treatment through immunotherapy [52,53].

It is interesting to note that while IDO1 seems to contribute to immune escape in cancer, its presence in interstitial foci in transplanted kidneys is associated with an increased risk of rejection. This suggests that the role of IDO1 might be context dependent, influenced by factors such as the local microenvironment [31,54,55,56]. Further research is needed to fully understand these complex interactions and their implications for cancer treatment and organ transplantation.

Our findings also highlight the potential of IDO1 as a nuanced biomarker for predicting and monitoring rejection episodes. It has been suggested as a potential biomarker in diabetic nephropathy and atopic dermatitis [57,58]. However, the exact predictive value and clinical utility of IDO1 as a biomarker in this context require further validation through more extensive studies and longitudinal analyses. It is also worth considering the broader implications of our findings in the context of immunosuppressive therapy. Given the differing roles of IDO1 in TE and interstitial foci, a one-size-fits-all approach to immunosuppression may not be optimal. Tailoring immunosuppressive strategies based on the specific patterns of IDO1 expression could enhance graft survival and patient outcomes.

Despite the promising findings, there is a significant need for further research to confirm and elucidate the precise role of IDO1 in kidney graft rejection. Future studies should focus on longitudinal analyses involving larger patient cohorts to validate IDO1 as a rejection biomarker and understand the underlying mechanisms by which IDO1 influences the immune response in kidney transplantation. Such research will clarify the role of IDO1 in transplant immunology, potentially leading to more effective and personalised treatment strategies.

### 4.1. Study Limitations

#### 4.1.1. Challenges in Immunohistochemistry Techniques

Immunohistochemistry (IHC) is a fundamental method in pathological analysis but presents technical hurdles [59,60]. Issues such as variations in tissue fixation quality, methods for antigen retrieval, and antibody specificity can lead to consistency in staining intensity and patterns. These factors may have influenced the interpretation of IDO1 expression in our study, potentially affecting the precision and dependability of our results.

#### 4.1.2. Interpretative Subjectivity

Immunohistochemistry (IHC) primarily yields qualitative data rather than quantitative data [61]. As a result, categorising biopsies as IDO1(+) positive based on any staining level introduces subjectivity. This methodological constraint might overlook subtle variations in IDO1 expression levels that could be critical for accurately correlating with transplant rejection outcomes.

#### 4.1.3. Variability in IDO1 Expression Dynamics

A clearer understanding of how IDO1 expression evolves within renal tubules is still necessary. The timing of IDO1 induction and its relationship with rejection episodes are complex and may not be fully captured by a single biopsy. This temporal variability introduces uncertainties that could impact the precision and dependability of linking IDO1 expression with rejection.

#### 4.1.4. Confounding Variables

Designating biopsies as IDO1-IF(+) positive, regardless of staining intensity, might miss potential confounding factors. IDO1 expression alone does not reveal the underlying biological mechanisms or functional consequences. External factors such as local inflammation or other tissue-specific conditions could obscure its association with rejection, possibly resulting in inaccurate interpretations.

#### 4.1.5. Future Research Directions

Our study highlights substantial gaps in understanding the presence and role of IDO1 in renal tubules. Addressing these gaps is crucial for improving the specificity and sensitivity of IDO1 as a biomarker for rejection. Future research should clarify the temporal dynamics and functional implications of IDO1 expression in relation to transplant rejection, paving the way for more accurate and reliable diagnostic and therapeutic strategies.

## 5. Conclusions

The study demonstrated that IDO1 expression in interstitial foci among patients with impaired renal transplants was associated with a higher incidence of rejection. Despite the challenges and limitations of this research, it enhances our understanding of the role of IDO1 in kidney transplantation and its potential as a biomarker for rejection. Furthermore, the correlation between IDO1 expression and rejection rates, particularly in TCMR and AMR, underscores its importance in evaluating immune-related damage. These findings highlight the potential of IDO1 as a marker and therapeutic target, offering promise for tailored treatments that could improve graft outcomes and long-term success in kidney transplantation.

## Figures and Tables

**Table 1:** Patient characteristics based on indoleamine 2,3-dioxygenase (IDO1) expression in interstitial foci are detailed, with the average ± standard deviation data.

Patient Attributes	IDO1 in Interstitial Foci IDO1-IF(+) Positive *n* = 80	IDO1 in Interstitial Foci IDO1-IF(−) Negative *n* = 41	*p*-Value
Recipient’s age at the time of transplantation (years)	41.0 ± 14.71	43.83 ± 14.29	0.31
Male gender (*n*, %)	53 (66.25%)	32 (78.0%)	0.18
Number of HLA ^1^ ABDR ^2^ mismatches	3.51 ± 1.41	3.61 ± 1.13	0.711
A	1.27 ± 0.60	1.34 ± 0.47	0.53
B	1.37 ± 0.60	1.47 ± 0.54	0.37
DR	0.83 ± 0.52	0.84 ± 0.58	0.88
Percentage of pre-sensitised patients			
PRA ^3^ <10%	33/45 (73.33%)	23/33 (69.7%)	0.72
PRA 10–50%	9/45 (20%)	8/33 (24.24%)	0.65
PRA >50%	3/45 (6.67%)	2/33 (6.06%)	0.91
Cold ischemia time (hours)	22.17 ± 7.79	22.03 ± 9.10	0.94
Donor male gender (*n*, %)	31/80 (38.75%)	22/41 (53.66%)	0.12
Donor age (years)	46.98 ± 16.08	50.0 ± 14.40	0.34
Delayed graft function	2/80 (2.5%)	2/41 (4.88%)	0.52

^1^ Human Leukocyte Antigen; ^2^ HLA-A, HLA-B (Class I) and HLA-DR (Class II)-specific classes of HLA genes; ^3^ Panel Reactive Antibody.

**Table 2:** The data include exact numbers and percentages for cases testing positive for a specific factor.

Immunosuppression	IDO1 Expression in Interstitial Foci IDO1-IF(+) Positive *n* = 80	IDO1 Expression in Interstitial Foci IDO1-IF(−) Negative *n* = 41	*p*-Value
Tacrolimus	55 (68.75%)	30 (73.17%)	0.61
Cyclosporin	25 (31.25%)	11 (26.83%)	0.61
MMF/MPA ^1^	80 (100%)	41 (100%)	NS
Azathioprine	2 (2.5%)	0 (0%)	NS
Anti-CD25 ^2^ therapy	1 (1.25%)	1 (1.25%)	0.63

^1^ Mycophenolate Mofetil or Mycophenolic Acid; ^2^ Interleukin-2 receptor alpha chain.

**Table 3:** Logistic regression analysis with different variables taken into consideration.

Variable	Coefficient	Standard Error	*p*-Value	Odds Ratio	95% Confidence Interval
IDO presence in IF	1.7464	0.4780	0.0003	5.7341	(2.2470, 14.6324)
Recipient’s sex ^1^	0.6064	0.4825	0.2088	1.8338	(0.7123, 4.7212)
HLA MM >3 ^2^	−0.7089	0.4334	0.1019	0.4922	(0.2105, 1.1509)
PRA >50% ^3^	0.0608	0.9684	0.9499	1.0627	(0.1593, 7.0909)
CIT >20 h ^4^	0.8753	0.4406	0.0469	2.3997	(1.0119, 5.6906)
Living donor	0.4948	1.0903	0.6499	1.6402	(0.1936, 13.8971)
Proteinuria > 0.5 g ^5^	−0.2138	0.4135	0.6050	0.8075	(0.3591, 1.8159)
DGF ^6^	0.4000	1.2219	0.6050	1.4918	(0.1360, 16.3618)
Glomerulonephritis	−0.3415	0.4410	0.4387	0.7107	(0.2994, 1.6869)
Tacrolimus	−1.3162	0.7772	0.0904	0.2682	(0.0585, 1.2302)
Cyclosporin	−0.8994	0.7818	0.2500	0.4068	(0.0879, 1.8830)
Constant	−0.5004	0.9398	0.5944		

^1^ For purposes of the analysis: 1 = male, 0 = female; ^2^ Human leukocyte antigen mismatches, more than 3; ^3^ Panel-reactive antibodies, more than 50%; ^4^ Cold ischemia time, more than 20 h; ^5^ Proteinuria larger than 0.5 g; ^6^ Delayed graft function.

**Table 4:** The table gives an overview of the findings and rejection types linked to indoleamine 2,3-dioxygenase (IDO1) expression in interstitial foci, detailing the incidence and proportions of positive and negative cases.

Biopsy Diagnosis	IDO1 Expression in Interstitial Foci IDO1-IF(+) Positive *n* = 80	IDO1 Expression in Interstitial Foci IDO1-IF(−) Negative *n* = 41	*p*-Value
Rejections (all)	46/80 (57.5%)	10/41 (24.34%)	0.0005
AMR ^1^ (including pure and mixed)	12/80 (15%)	1/41 (2.44%)	0.035
TCMR ^2^ (including pure and mixed)	43/80 (53.75%)	7/41 (17.07%)	0.0001
AMR (pure)	2/80 (2.5%)	1/41 (2.44%)	0.98
TCMR (pure)	33/80 (41.25%)	7/41 (17.07%)	0.007

^1^ Antibody-mediated rejection; ^2^ T-cell-mediated rejection.

## Data Availability

The data presented in this study are available on request from the corresponding authors. The data are not publicly available due to privacy policies.

## Abbreviations

The following abbreviations are used in this manuscript:

IDO1	indoleamine 2,3-dioxygenase 1
IF	interstitial foci
HLA antibody	human leukocyte antigen antibody
non-HLA antibody	non-human leukocyte antigen antibody
PRA	panel reactive antibody
AMR	antibody-mediated rejection
TCMR	T-cell-mediated rejection
MMF	mycophenolate mofetil
MPA	mycophenolic acid
